# The complete chloroplast genome of the *Euphorbia maculata* L. (Euphorbiaceae): characterization and phylogeny

**DOI:** 10.1080/23802359.2020.1833778

**Published:** 2020-11-06

**Authors:** Yiheng Wang, Jun Luo, Mengli Wang, Yang Ge, Lanping Guo

**Affiliations:** a National Resource Center for Chinese Meteria Medica, China Academy of Chinese Medical Sciences, Beijing, China;; b Natural Resources Bureau of Xishan District of Kunming City, Kunming City, People’s Republic of China

**Keywords:** *Euphorbia maculata*, chloroplast genome, phylogenetic analysis

## Abstract

*Euphorbia maculata* is an important medicinal plants of the family Euphorbiaceae. The complete chloroplast genome reported here is 162,685 bp in length, including two inverted repeats (IRs) of 26,822 bp, which are separated by a large single-copy (LSC) and a small single-copy (SSC) of 90,514 bp and 18,527 bp, respectively. The whole chloroplast genome of *E. maculata* contains 111 genes, including 77 protein-coding genes, 4 transfer RNA, and 30 ribosome RNA. Phylogenetic analysis indicated that *E. maculata* is closely related to *E. milii* and *E. tirucalli.*

*Euphorbia maculata* L. (Spotted spurge) is a fast-growing annual weed native to eastern North America and spread to Asia, Africa and Europe (Wu et al. 1994). It functions as a pioneer species with a prostrate growth habit in ecological succession. The milky sap of the plant is an irritant for many people. Like many members of the family Euphorbiaceae, it has been widely used as a folk medicine, which can produce anti-inflammatory and cancer chemopreventive agents of triterpenoids (Yi et al. 2018). It is necessary to develop genomic resources for *E. maculata* to provide intragenic information for its utilization and chloroplast genomes are valuable sources (Dong et al. 2020; Sun et al. 2020).

The fresh leaves of *E. maculata* were collected from Luxi county, Hunan province, China (28°12’59”N, 110°13’11”E). Voucher specimens were deposited in Institute of Chinese Materia Medica (Specimen accession number: 430723LY0485), China Academy of Chinese Medical Sciences. Total genomic DNA was extracted with the modified cetyltrimethyl ammonium bromide (CTAB) method (Li et al. 2013). Paired-end libraries were prepared with the NEBNext Ultra DNA Library Prep Kit. The genome was sequenced using the HiSeq X Ten platform (Illumina, Santiago, CA, USA). All good quality paired reads were assembled using the Spades program to contigs (Bankevich et al. 2012). Chloroplast genome sequence contigs were selected by the program BLAST (Altschul et al. 1990) using *E. milii* (Genbank accession number: MN713924) as a reference and the selected contigs were assembled using Sequencher 4.10 (Gene Codes Corporation, Ann Arbor, MI USA, http://www.genecodes.com). Chloroplast genome annotation was performed with Plann (Huang and Cronk 2015) using the *E. milii* as reference sequence. The annotated sequence was submitted to the GenBank under the accession number MT830858.

The complete chloroplast genome reported here is 1,62,685 bp in length, including two inverted repeats (IRs) of 26,822 bp, which are separated by a large single-copy (LSC) and a small single-copy (SSC) of 90,514 bp and 18,527 bp, respectively. The overall GC-content of the chloroplast genome was 35.4%. The chloroplast DNA of *E. maculata* comprised 111 distinct genes, including 77 protein-coding genes, 4 transfer RNA, and 30 ribosome RNA, but didn’t contain *cemA* and *rpl22* these two protein-coding genes. In these genes, 17 harbored a single intron, while two (*ycf3*and *clpP*) contained double introns.

In order to confirm the phylogenetic relationships of *E. maculata* within the genus *Euphorbia* and other related groups, total 26 complete cp genomes were obtained from Genbank and the genus *Aristolochia* was taken as an outgroup. All chloroplast genome sequences were aligned using MAFFT (Katoh et al. 2019) and ambiguous alignment regions were trimmed by Gblocks (Castresana 2000). Phylogenetic analysis was conducted based on maximum-likelihood (ML) analyses using RAxML (Stamatakis 2014), under the GTR + G model with 1000 rapid bootstrap replicates. The phylogenetic tree showed that all species of *Eupho*rbia form a monophyletic group with 100% support, and *E. maculata* is closely related to *E. milii* and *E. tirucalli* (Figure 1). The chloroplast genome of *E. maculata* provided a lot of genetic information for species conservation and identification of genus *Eupho*rbia.

**Figure 1.**
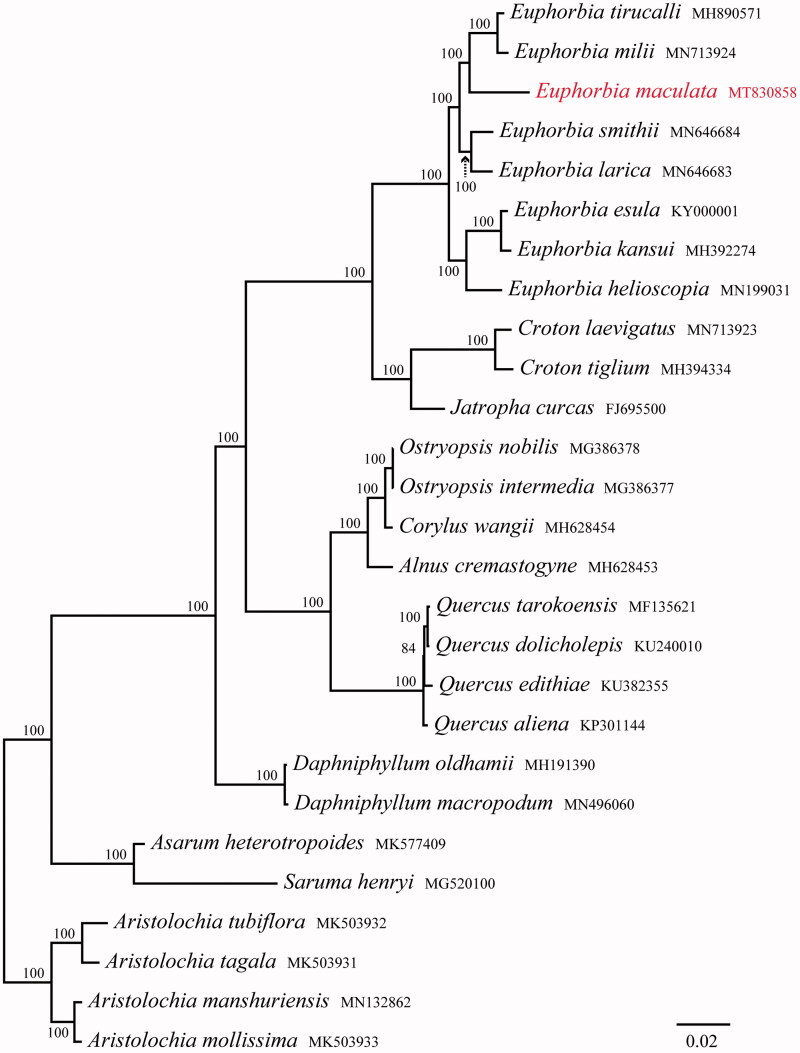
Phylogenetic tree reconstruction of 27 taxa using maximum likelihood (ML) methods in the chloroplast genome sequences. ML bootstrap support value presented at each node.

## Data Availability

The data that support the findings of this study are openly available in GenBank of NCBI https://www.ncbi.nlm.nih.gov/, reference number MT830858, raw data BioProject ID: PRJNA662166.
